# Left displacement of the third gastric compartment in an alpaca: the first case report in China

**DOI:** 10.1186/s12917-022-03181-z

**Published:** 2022-03-04

**Authors:** Yuxi Song, Zheng Wang, Ran Li, Dandan Hao, Zhijie Wang, Cheng Xia, Yunlong Bai

**Affiliations:** College of Animal Science and Veterinary Medicine, Heilongjiang Bayi Agricultural University, Daqing, China

**Keywords:** Alpaca, Abdominal distension, LDC3, Surgery

## Abstract

**Background:**

Left displacement of the third gastric compartment (LDC3) in alpacas is an extremely rare condition and has not been reported thus far. Therefore, we describe the clinical diagnosis and treatment of LDC3 in an alpaca.

**Case presentation:**

A 2-year-old brown female alpaca (*Vicugna pacos*) was presented to evaluate a 3-day history of abdominal distension causing loss of both appetite and thirst, along with oliguria and low to no defecation. Clinical examination, X-ray examination, surgical exploration, and determination of gastric pH (pH ~ 2.35) confirmed that LDC3 resulted in abdominal distension. The gastric wall of the displaced third gastric compartment was incised for the expulsion of pneumatosis, and a medical-grade silicone tube was inserted into the incision to remove the effusion by siphoning. Surgical treatment proved to effectively alleviate the abdominal distension caused by LDC3 without apparent side effects.

**Conclusions:**

To our knowledge, this case is the first known report of LDC3 in an alpaca in China. A similar condition, left displaced abomasum, has previously been described in cattle and sheep.

## Background

Since its initial identification in 1950, left displaced abomasum (LDA) has been increasingly observed in cattle [1, 2]. Such disease also occurs occasionally in sheep and goats [3, 4]. It is characterized by an abomasum filled with gas floating in the dorsal part of the left abdomen, with an acute onset and high mortality [2]. Left untreated, LDA generally results in adhesions between the abomasal wall and other organs, multiple organ failure, and even death [5]. Patients eventually succumb to cachexia or abomasal perforation-induced peritonitis [5].

Alpaca (*Vicugna pacos*), a member of the South American Camelids (SACs) family, belongs to the suborder Tylopoda of the order Artiodactyla, whose gastric anatomy is adapted to the physiological process of rumination and the degradation of plant cell wall contents to a great extent [6]. However, unlike the four-chambered stomach of ruminants such as cattle and sheep, alpacas have three gastric compartments. The specific design of the stomach together with its related functions and physiological processes confirm that the evolution of Tylopoda and Ruminantia took place in parallel and not in homology [6].

Despite the uniqueness in anatomical structures and physiological function, the third gastric compartment (C3) in alpacas is similar to the abomasum of cattle and sheep in functions such as the secretion of gastric juices and food digestion [7]. Thus, in many respects, left displacement of the third gastric compartment (LDC3) in alpacas may be similar to LDA in cattle and sheep. Unexpectedly, unlike the high incidence of LDA in cattle, LDC3 in alpacas is an extremely rare condition and has not been reported thus far [8]. Notably, this is the first alpaca LDC3 case found in clinical practice in China. Therefore, we describe the clinical diagnosis and treatment of LDC3 in an alpaca.

### Case presentation

A 2-year-old, 58 kg brown female alpaca was presented to the Animal Hospital of Heilongjiang Bayi Agricultural University (Daqing, China) to evaluate abdominal distension. For ornamental purposes, the alpaca was purchased into a sighting agricultural park of Daqing in Heilongjiang Province in China. The alpaca was fasted on the day of purchase but allowed free access to water. On Day 3, it was fed large quantities of carrots and a small amount of alfalfa green grass. During this period, the alpaca presented with unformed stool followed by loss of both appetite and thirst, along with oliguria and low to no defecation. The sick alpaca was treated in our hospital on Day 6.

**Fig. 1:**
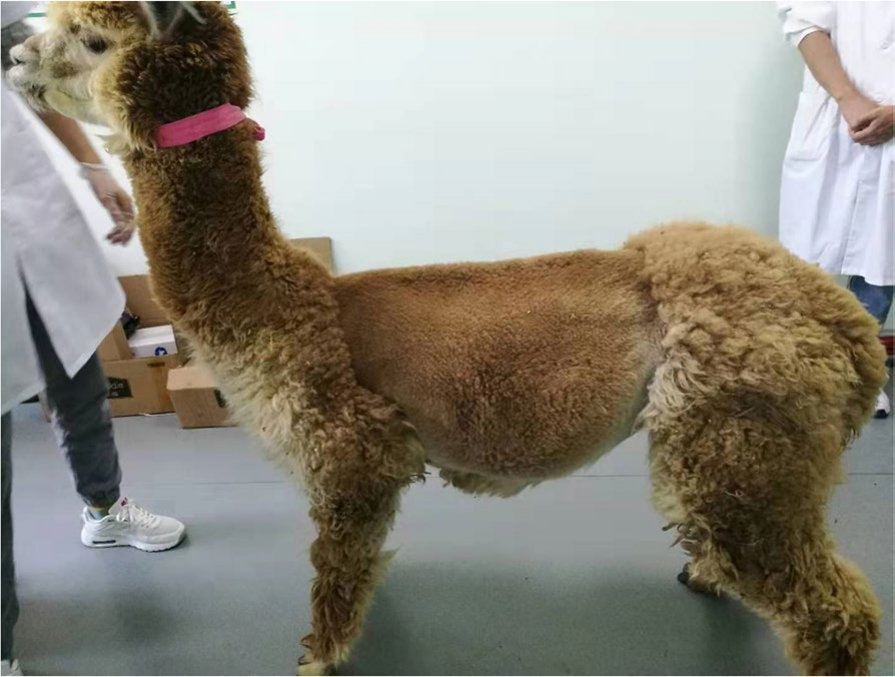
Left abdominal view in a 2-year-old brown female alpaca with LDC3 after shaving. The image showed a marked abdominal distension

On admission, the alpaca exhibited overall malnutrition and abdominal distension (Fig. 1). The rectal temperature [38.6 °C, reference interval (RI): 37.5 °C to 38.9 °C], heart rate (60 beats/min, RI: 60 to 90 beats/min), and respiratory rate (30 breaths/min, RI: 10 to 30 breaths/min) were within the reference ranges of SACs [9]. There was a high-pitched metallic “ping” and splash auscultated upon percussion and succussion of the left abdomen, suggesting that there could be effusion and pneumatosis in the first gastric compartment (similar to the rumen of cattle and sheep) [7] or abdominal cavity.

**Fig. 2:**
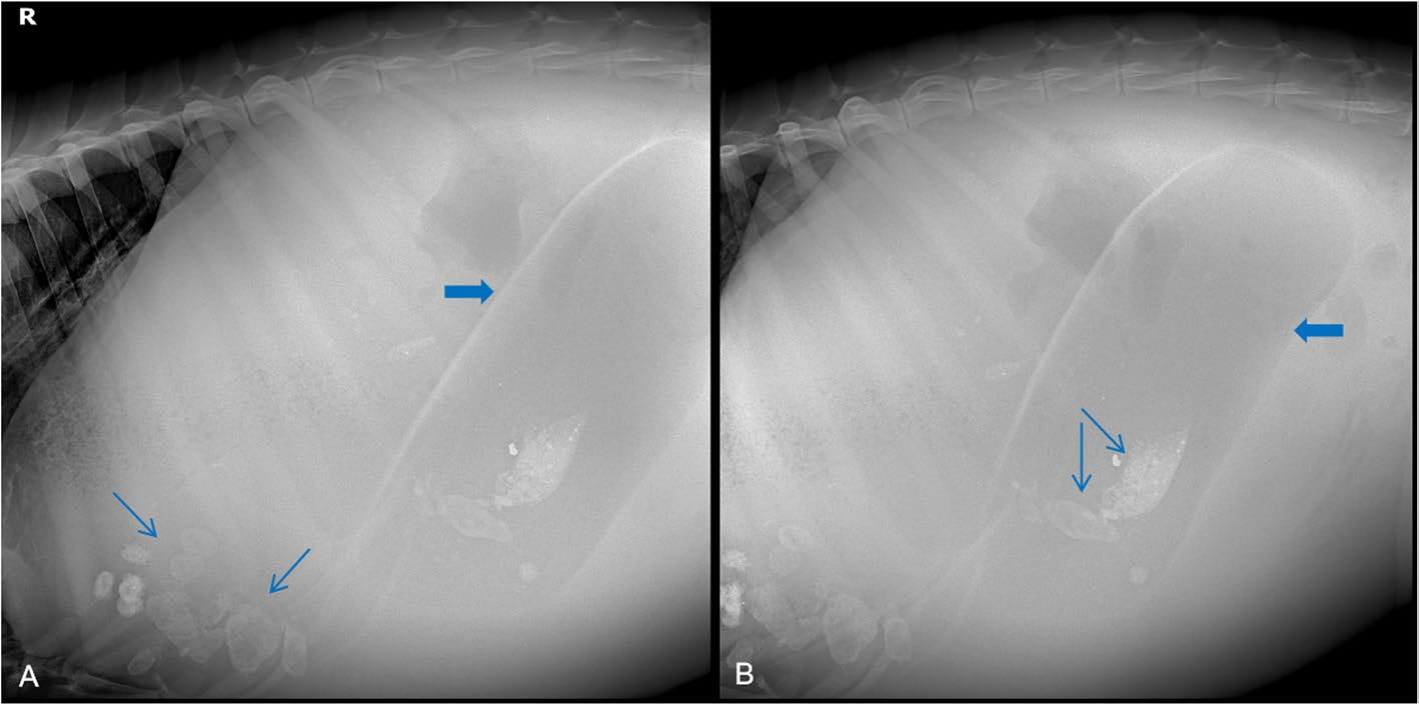
Standing left lateral abdominal X-ray images of the alpaca. **A** X-ray showed an oval, tubular, low-density shadow (thick arrow) and multiple shadows (thin arrows) with different sizes and densities. **B** X-ray showed a whole C3 (thick arrow) and two higher densities of diamond shadings (thin arrows)

Abdominal X-ray showed an oval, tubular, notably demarcated, low-density shadow (suggestive of effusion and pneumatosis) in the middle portion of the left posterior abdomen (Fig. 2A). The patient was suspected to have left displacement of the third gastric compartment. In the lower third of the shadow, there were two higher densities of diamond shadings (Fig. 2B). In addition, there were multiple shadows with different sizes and densities in the anterior left ventral down that were suspected to be stones or other foreign bodies (Fig. 2A).

**Fig. 3:**
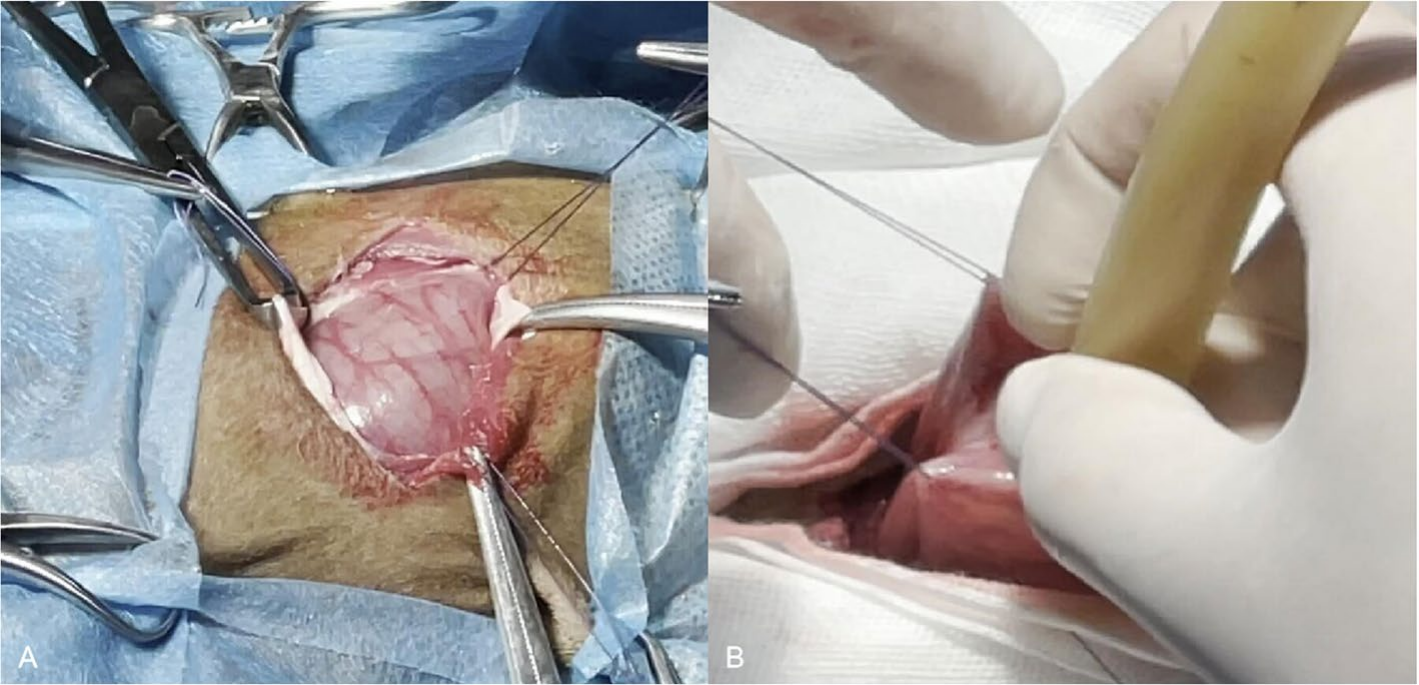
Surgical exploration (**A**) and expulsion of the pneumatosis and effusion (**B**). **A** Appearance of C3 was exposed after left abdominal wall incision. **B** A medical-grade silicone tube was inserted into the C3 incision to release the pneumatosis and effusion

Surgery was performed as previously described for LDA in cattle [10]. Briefly, peripheral venous access was established, and prophylactic antibiotics were given with intravenous cefoxitin 1 g within 30 min before the beginning of surgery. The left paralumbar fossa was shaved, and the skin was disinfected with 2% iodine tincture and 75% ethanol. Local anaesthesia was then carried out with 5 ml of 1% lidocaine hydrochloride by using a paravertebral nerve block. To meet the owner’s requirements, a 3–5 cm longitudinal skin incision was made 2 cm below the centre of the left inguinal fossa under the premise of guaranteeing aesthetics. The abdominal wall musculature (external oblique, internal oblique, transversus abdominis, and rectus abdominis) was excised by blunt dissection. This was followed by an incision through the peritoneum to expose the displaced C3 (Fig. 3A). C3 was long and tubular by surgical exploration, and a small amount of clear ascites was observed in the abdominal cavity.

Next, a gastric wall incision of approximately 1 cm was made to release gas. A medical-grade silicone tube was inserted into the incision to reduce abdominal bloating and drain most of the contents appeared yellowish-brown and muddy in appearance, with a rancid odour (Fig. 3B). The pH of the gastric contents was 2.35, as measured using an acidometer. The gastric mucosa was pale pink. Therefore, a diagnosis of LDC3 was confirmed by clinical examination, X-ray examination, surgical exploration, and gastric content pH detection. Subsequently, right reduction was performed without fixation. Immediately after that, the peritoneum, muscles, and skin were sutured in turn, and the surgery area was sterilized with a 2% iodine tincture. A variable infusion of 0.9% normal saline (250 mL) and 5% dextrose (250 mL) was given intravenously, and cefquinome (5 mg/kg body weight) was administered by subcutaneous injection. The alpaca was allowed to stand, and no abnormalities were observed. Ultimately, the alpaca was returned to the park for usual care and treatment under the guidance of a resident veterinarian. Six months later, the owner reported that the alpaca was growing well and had experienced no complications.

## Discussion and conclusions

The aetiology of LDA in cattle is multifactorial [11]. Abomasal atony and excessive gas production have been reported as prerequisites for the development of LDA [12]. The exact reasons for LDA are unknown, yet long-distance transportation, appetite suppression, changes in feed, and metabolic stress and disorders have been associated with this disorder [5, 13, 14]. In our case, the alpaca was started on a fasting regimen after being purchased. In addition, the purchasing process involved long-distance transportation. Subsequently, the alpaca was fed large quantities of carrots and a small amount of alfalfa green grass, and clinical symptoms appeared 2 days later. Therefore, it is reasonable to speculate that the above factors could be associated with the onset of LDC3 in an alpaca. However, the specific reasons still need further investigation in clinical practice and animal experiments.

The diagnosis of LDA in cattle is based on clinical signs and auscultation and percussion of the abdomen. As previously reported for LDA in cattle, the alpaca with LDC3 in this case report had a normal rectal temperature, heart rate and respiratory rate [15, 16]. As evidence for the presence of abomasal displacement, clinical signs of indigestion, reduced ruminal movements, inappetence, and ping and splashing sounds on the side of displacement have been previously reported in cattle and sheep [17, 18]. Coincidentally, the alpaca with LDC3 reported here presented with clinical signs similar to those in cattle and sheep with LDA. In addition, the alpaca had left-sided abdominal distention and a left-sided pinging and splash were auscultated on percussion and succussion. The same diagnostic approaches may therefore be applicable for SACs with LDC3. However, finding abdominal distension in an alpaca is not pathognomonic for LDC3. Other differentials for abdominal distension in an alpaca include impaction, tympany and acidosis of the first gastric compartment, traumatic peritonitis of the second gastric compartment, and third gastric compartment ulcers [19–21]. Therefore, LDC3 in SACs should be distinguished clinically from these diseases.

In the case reported here, abdominal X-ray examination was a simple, non-invasive approach of confirming a clinical suspicion of LDC3 and monitoring the position of displaced C3. The normal radiographic appearance of C3 in alpacas has been described previously [22]. Imaging the abomasum dorsally between the rumen and left abdominal wall in conjunction with medial displacement of the rumen has been reported as radiographic evidence of LDA in cattle [23, 24]. Similar radiographic abnormalities were readily identifiable in the alpaca reported here and accurately identified LDC3. Given its practicability of diagnosing LDC3 in alpacas, X-ray examination may obviate the need for surgical exploration in cases with subtle clinical signs. However, the identities of two higher densities of the diamond shadings in the lower third of C3, as well as the multiple shadows with different sizes and densities at the anterior left ventral down, are unclear. Therefore, further pathoanatomical studies are needed to identify whether these shadows are related to the occurrence of LDC3.

In the alpaca described here, surgical exploration supported a clinical suspicion of LDC3. Abdominal exploration revealed no anatomic abnormalities or adhesions and concomitant or concurrent disorders. The determination of gastric pH also supported a clinical suspicion of LDC3. In addition, hypochloremic and hypokalemic metabolic alkalosis is the most common metabolic change in cattle diagnosed with LDA. Blood gas and electrolyte analysis have been used to support a clinical suspicion of LDA in previous studies [17, 25]. Based on these prior studies, identifying hypochloremia, hypokalaemia, and metabolic alkalosis could be useful for the diagnosis of LDC3 in alpacas with subtle clinical signs. Regrettably, data on blood gas and chemistry from the alpaca with LDC3 could not be made available because the owner elected not to have any further testing performed. This is also the major limitation of our case report, and future research should consider this clinical aspect.

Surgery to correct abomasal displacements likely accounts for the majority of nonelective surgical procedures in cattle but is uncommon in small ruminants [26]. Multiple surgical techniques have been described for managing LDA in calves, including left paralumbar fossa abomasopexy, right paralumbar omentopexy, right paracostal abomasopexy, and right paramedian abomasopexy [27, 28]. Alpacas are likely to lie down during surgery, so a recumbent technique (left flank laparotomy) was chosen. As an ornamental animal, the alpaca received a smaller skin incision to fulfill the breeders’ requirements. In general, cows with LDA have a good prognosis for returning to a productive life after surgical replacement. Likewise, in our case report, the alpaca with LDC3 was successfully cured. As expected, the owner was satisfied with the cosmetic appearance of the abdominal skin. This successful case suggested that surgical treatment could also be suitable for SACs with LDC3.

Our case highlights the diagnosis and management of previously unreported LDC3 in an alpaca presenting with overall malnutrition and abdominal distension. Left displacement of the third gastric compartment should be considered in the differential diagnosis for SACs with abdominal distention. The combination of clinical examination, X-ray examination, surgical exploration, and gastric content pH detection is useful in the diagnosis of LDC3. Surgical correction is highly effective for treating LDC3 in alpacas. Importantly, this is the first case report of alpaca LDC3 in China, which aims to further improve the knowledge of LDC3 in a previously unreported species and to help veterinarians diagnose and treat this disease by borrowing the clinical approaches to LDA in cattle and sheep. Further research on the blood clinicopathological features and pathoanatomical structures of LDC3 in SACs is warranted to better understand the pathology and treatment of C3 in these species.

## Data Availability

Not applicable.

## Abbreviations

C3: Third gastric compartment
LDA: Left displaced abomasum
LDC3: Left displacement of the third gastric compartment
RI: Reference interval
SACs: South American camelids
